# Prevalence of non *Helicobacter pylori *species in patients presenting with dyspepsia

**DOI:** 10.1186/1471-230X-12-3

**Published:** 2012-01-06

**Authors:** Javed Yakoob, Zaigham Abbas, Rustam Khan, Shagufta Naz, Zubair Ahmad, Muhammad Islam, Safia Awan, Fatima Jafri, Wasim Jafri

**Affiliations:** 1 Department of Medicine, The Aga Khan University, Karachi, Pakistan; 2 Department of Pathology, The Aga Khan University, Karachi, Pakistan; 3 Department of Community Health Sciences, The Aga Khan University, Karachi, Pakistan

**Keywords:** Dyspepsia, gastric biopsies, *H. pylori*, *H. heilmannii*, *H. felis*, coinfection, cats, dogs

## Abstract

**Background:**

Helicobacter species associated with human infection include *Helicobacter pylori, Helicobacter heilmannii *and *Helicobacter felis *among others. In this study we determined the prevalence of *H. pylori *and non-*Helicobacter pylori *organisms *H. felis and H. heilmannii *and analyzed the association between coinfection with these organisms and gastric pathology in patients presenting with dyspepsia. Biopsy specimens were obtained from patients with dyspepsia on esophagogastroduodenoscopy (EGD) for rapid urease test, histology and PCR examination for Helicobacter genus specific 16S rDNA, *H. pylori *phosphoglucosamine mutase (*glmM*) and urease B (*ureB*) gene of *H. heilmannii *and *H. felis*. Sequencing of PCR products of *H. heilmannii *and *H. felis *was done.

**Results:**

Two hundred-fifty patients with dyspepsia were enrolled in the study. The mean age was 39 ± 12 years with males 162(65%). Twenty-six percent (66 out of 250) were exposed to cats or dogs. PCR for Helicobacter genus specific 16S rDNA was positive in 167/250 (67%), *H. pylori glmM *in 142/250 (57%), *H. heilmannii *in 17/250 (6%) and *H. felis *in 10/250 (4%), respectively. All the *H. heilmannii *and *H. felis *PCR positive patients were also positive for *H. pylori *PCR amplification. The occurrence of coinfection of *H. pylori *and *H. heilmannii *was 17(6%) and with *H. felis *was 10(4%), respectively. Only one out of 66 exposed to pets were positive for *H. heilmannii *and two for *H. felis*. Histopathology was carried out in 160(64%) of 250 cases. Chronic active inflammation was observed in 53(56%) (p = 0.001) of the patients with *H. pylori *infection alone as compared to 3(37%) (p = 0.73) coinfected with *H. heilmannii *and *H. pylori *and 3(60%) coinfected with *H. felis *and *H. pylori *(p = 0.66). Intestinal metaplasia was observed in 3(3%)(p = 1.0) of the patients with *H. pylori *infection alone as compared to 2(25%) (p = 0.02) coinfected with *H. heilmannii *and *H. pylori *and 1(20%) coinfected with *H. felis *and *H. pylori *(p = 0.15).

**Conclusion:**

The prevalence of *H. heilmannii *and *H. felis *was low in our patients with dyspepsia. Exposure to pets did not increase the risk of *H. heilmannii *or *H. felis *infection. The coinfection of *H. pylori *with *H. heilmannii *was seen associated with intestinal metaplasia, however this need further confirmation.

## Background

*Helicobacter *species infect the gastrointestinal tracts of many animals from birds through humans. Some of these have been linked to a range of human diseases [1,2] including chronic gastritis, peptic ulcer disease, mucosa-associated lymphoid tissue lymphoma, and gastric adenocarcinoma [1,3]. The principal Helicobacter infection in humans is *Helicobacter pylori*, with infection rates in developing countries reaching 50% to 90% [2,4]. Human gastric biopsy samples, however, have shown to harbor bacteria which were morphologically different from *H. pylori *[5,6]. These include *Helicobacter heilmannii *and *Helicobacter felis *which are primarily pathogens of domestic animals and were later found to infect humans as well [7-9].

Gastric non-*Helicobacter pylori *helicobacters constitute a diverse group of bacterial species that are known to colonize the gastric mucosa of several animals [10]. These include morphologically distinct, typically long spiral shaped bacteria originally referred to as *Gastrospirillum hominis *and later as *H. heilmannii*. The latter was further subdivided in two taxa, types 1 and 2 [10]. *H. heilmannii *type 1 are identical to *H. suis *which colonizes the stomachs of pigs. The former *H. heilmannii *type 2 represent a group of species, known to colonize the gastric mucosa of dogs and cats and include *H. felis, H. bizzozeronii, H. salomonis, H. cynogastricus, H. baculiformis *and a bacterium provisionally named in 2004 as ''*Candidatus *H. heilmannii'' because at that time, it could not be cultured in vitro [10,11]. However, recently, in vitro cultures have been obtained resulting in description of *H. heilmannii*, as a novel Helicobacter species [12]. Sequencing of the 16S or 23S rRNA-encoding genes allows differentiation of *H. suis *from the other gastric non-*H. pylori *helicobacters species, but it cannot distinguish between *H. felis, H. bizzozeronii, H. salomonis, H. cynogastricus, H. baculiformis *and *Candidatus *H. heilmannii [10]. For differentiation between these species, sequencing of the heat shock protein 60 (hsp60) or gyrase B (gyrB) gene is used while sequencing of the urease A and B genes is considered to be the most suitable method since sequences of these genes are available [10,11,13,14].

Dyspepsia describes a variety of symptoms, including abdominal pain, bloating, nausea, and vomiting. In these patients, endoscopy is considered to rule out gastroesophageal reflux disease, peptic or duodenal ulcer and gastric cancer. The role of *H. pylori *infection in dyspepsia remains controversial. This study aims to identify the prevalence of *H. pylori *and non-H. pylori helicobacters, *H. felis *and *H. heilmannii *and to analyze the gastric pathology associated with coinfection of these organisms in patients presenting with dyspepsia.

## Results and discussion

Majority of the patients with *H. pylori *infection were in the age range of 18-39 years, while *H. felis *and *H. heilmannii *positive patients did not show this distribution. (Table 1). There was no difference in the gender, ethnicity of patients, crowding index (CI) and source of water distribution among the patients with *H. pylori *and non-*H. pylori *infections (Table 1). All patients had abdominal pain with endoscopic gastritis as the predominant finding. The false positive and false negative results obtained with RUT were 15(36%) and 6(12%), respectively while with histology the false positive and false negative results obtained were 20(30%) and 10(11%), respectively (Table 1-2).

**Table 1:** Demography and clinical features of patients enrolled

	PCR for *H. pylori*			PCR for *H. heilmannii*			PCR for *H. felis*		
	Positive n = 142	Negative n = 108	P value	Positive n = 17	Negative n = 233	P value	Positive n = 10	Negative n = 240	P value
**Age**									
18-39 years	81(57)	55(51)		8(47)	128(55)		6(60)	130(55)	
40-55 years	53(37)	35(32)	0.02	7(41)	81(35)	0.82	3(30)	85(35)	0.93
56-75 years	8(6)	18(17)		2(12)	24(10)		1(10)	25(10)	
**Gender**									
Male	98(69)	64(59)	0.11	11(65)	151(65)	0.99	5(50)	157(65)	0.33
Female	44(31)	44(41)		6(35)	82(35)		5(50)	83(35)	
**Ethnicity**									
Karachiite	36(25)	36(33)		4(24)	68(29)		2(20)	70(29)	
Quetta resident	47(36)	31(25)	0.15	5(29)	73(31)	0.81	3(30)	75(31)	0.75
Afghan	55(39)	45(42)		8(47)	92(40)		5(50)	95(40)	
**Crowding Index (CI)**									
0-1(low)	59(41)	39(36)		5(29)	93(40)		5(50)	93(39)	
2-4 (moderate)	82(58)	62(57)	0.03	12(71)	132(57)	0.35	5(50)	139(58)	0.59
> 4 (crowding)	1(1)	7(7)		0(0)	8(3)		0(0)	8(3)	
**Water supply**									
Municipal	86(61)	59(55)	0.34	8(47)	137(59)	0.34	7(70)	138(58)	0.52
Boring Water	56(39)	49(45)		9(53)	96(41)		3(30)	102(42)	
**EGD**									
Gastritis	136(96)	106(96)	0.47	17(100)	225(97)	1	10(100)	232(97)	1
Duodenal ulcer	6(4)	2(4)		0(0)	8(3)		0(0)	8(3)	
**Rapid Urease test (n = 90**)									
Positive	42(88)	15(36)	> 0.001	4(44)	53(63)	0.28	2(40)	55(63)	0.35
Negative	6(12)	27(64)		5(56)	28(37)		3(60)	30(37)	
**Histopathology (n = 160)**									
Bacterial density									
Occasional	10(11)	40(61)		3(37)	47(31)		0(0)	50(32)	
Few in some fields	59(63)	21(32)	> 0.001	2(25)	78(51)	0.27	4(80)	76(49)	0.14
Only 1/2 small clusters	25(27)	5(7)		3(38)	27(18)		1(20)	29(19)	
Inflammation type									
Chronic	41(44)	47(71)	0.001	5(63)	83(55)	0.73	2(40)	86(55)	0.66
Chronic active inflammation	53(56)	19(29)		3(37)	69(45)		3(60)	69(45)	
Lymphoid follicles									
Positive	14(15)	8(12)	0.73	0(0)	22(14)	0.30	0(0)	22(14)	0.47
Negative	80(85)	58(88)		8(100)	130(86)		5(100)	133(86)	
Intestinal metaplasia									
Positive	3(3)	2(3)	1.0	2(25)	3(2)	0.02	1(20)	4(3)	0.15
Negative	91(97)	64(97)		6(75)	149(98)		4(80)	151(97)	

Univariate analysis was performed by using the independent sample t-test, Pearson Chi-square or Fisher Exact test where appropriate. A *P*-value of < 0.05 was considered as statistically significant. *All the *H. heilmannii *and *H. felis *PCR positive patients were also positive for *H. pylori *PCR amplification.

**Table 2:** PCR results for Helicobacter species

	PCR for Helicobacter genus specific *16SrRNA*		
	**Positive n = 167**	**Negative n = 83**	**P value**

***H. pylori glmM***			
Positive	133(80)	9(11)	< 0.001
Negative	34(20)	74(89)	
***H. heilmannii ureB***			
Positive	17(10)	0(0)	0.003
Negative	150(90)	83(100)	
***H. felis ure A and B***			
Positive	10(6)	0(0)	0.03
Negative	157(94)	83(100)	

Univariate analysis was performed by using the independent sample t-test, Pearson Chi-square test or Fisher Exact test where appropriate. A *P*-value of < 0.05 was considered as statistically significant. *All the *H. heilmannii *and *H. felis *PCR positive patients were also positive for *H. pylori *PCR amplification.

PCR for Helicobacter genus specific *16S rDNA *was positive in 167/250 (67%), *glmM *(*H. pylori*) in 142/250 (57%), *H. heilmanii *in 17/250 (6%) and *H. felis *in 10/250 (4%), respectively (Table 2).

PCR was positive for both *H. pylori *and *H. heilmannii *in 17(6%) and for *H. pylori *and *H. felis *in 10(4%), respectively (Table 2). All the *H. heilmannii *and *H. felis *positive patients were also positive for *H. pylori glmM *PCR amplification (Table 2).

26% (66 out of 250) were exposed to pets either cats or dogs. Most *H. heilmannii *positive patients did not have pet contact. Only one out of 66 exposed to pets was positive for *H. heilmannii *and two for *H. felis *(Table 3).

**Table 3:** Association of Helicobacter species with pets

	PCR for *H. pylori*			PCR for *H. heilmannii*			PCR for *H. felis*		
	**Positive n = 142**	**Negative n = 108**	**P value**	**Positive n = 17**	**Negative n = 233**	**P value**	**Positive n = 10**	**Negative n = 240**	**P value**

**Pets**									
Yes	42(30)	24(22)	0.19	1(6)	65(28)	0.05	2(20)	64(27)	1
No	100(70)	84(78)		16(94)	168(72)		8(80)	176(73)	

Univariate analysis was performed by using the independent sample t-test, Pearson Chi-square test or Fisher Exact test where appropriate. A *P*-value of < 0.05 was considered as statistically significant. *All the *H. heilmannii *and *H. felis *PCR positive patients were also positive for *H. pylori *PCR amplification.

A higher degree of bacterial density was associated with *H. pylori *infection alone (p < 0.001) (Table 1). Chronic active inflammation was observed in 53(56%) cases with *H. pylori *alone infection (p = 0.001) compared to 3(37%) in *H. heilmannii *(p = 0.73) and 3(60%) in *H. felis *positive patients coinfected with *H. pylori *(p = 0.66) (Table 1). Intestinal metaplasia (IM) was present in 3(3%) out of 94 cases with *H. pylori *infection alone compared to 2(25%) out of 8 cases of *H. Heilmannii *and *H. pylori *coinfection, and 1(20%) out of 5 cases of *H. felis *and *H. pylori *coinfection in which histology has been performed.

PCR product sequences were compared to the sequences of ureaseB of different *H. heilmannii *and *H. felis *strains. The *H. heilmannii *sequences had 100% similarity to '*Candidatus *Helicobacter heilmannii' strains GenBank: AF508012 and L25079; while it was 99% to GenBank: AY139170, AF507996, AY139172, AY139173 and 98% to GenBank: AY139171, respectively. The *H. felis *sequences had 100% similarity to *H. felis *strains GenBank: FQ670179 and X69080; while it was 99% to *H. felis *GenBank: AY368267, AY368261 and 98% to GenBank: DQ865138, respectively.

Among our patients, the cohort exposed to pet animals was limited to 26%. There were more patients with *H. pylori *infection who were in the 18-39 years age range. Such age distribution was not seen in cases with *H. felis *and *H. heilmannii *infection. There was no difference in the gender, ethnicity of patients, crowding index (CI) and source of water distribution among the patients with *H. pylori *and non-*H. pylori *helicobacter species infections. There were no statistically significant differences in the endoscopic findings in patients with *H. pylori *infection alone or with coinfection of *H. pylori *and non-*H. pylori *Helicobacter species. Chronic active inflammation was associated with *H. pylori *infection compared to *H. heilmannii *or *H. felis *coinfections with *H. pylori *(Table 1). However, the histology was not obtained in all the cases that showed *H. heilmannii *and *H. felis *infection. Intestinal metaplasia was present in 2(25%) out of 8 cases of *H. heilmannii *coinfection with *H. pylori *and in 1(20%) out of 5 cases of *H. felis *coinfection with *H. pylori *as compared to 3(3%) of 94 cases with *H. pylori *infection alone who had undergone the histological study. Although it was not possible to draw a conclusion that IM was significantly associated with the coinfection of either of the species and *H. pylori*, a tendency in that way would be likely, as it has also been reported by other authors.

PCR positives at the species level were also positive for the Helicobacter genus specific 16S rDNA and all the *H. heilmannii *and *H. felis *positive patients were also positive for *H. pylori *glmM PCR (Table 2). PCR product sequences of ureaseB gene of *H. heilmannii *had shown 100% similarity to '*Candidatus *H. heilmannii strains' GenBank: AF508012 and L25079; while *H. felis *sequences had shown 100% similarity to strains GenBank: FQ670179 and X69080.

In this study, we used urease gene-based PCR method developed by Nieger et al that detected only '*Candidatus *H. heilmannii' DNA from pure in vitro cultures of other non-*H. pylori *helicobacter species [14]. This method was also used by other investigators to demonstrate the presence of *Candidatus *H. heilmannii DNA in gastric biopsies from patients with dyspepsia [11,15,16]. The limitations of our study include the small number of patients who had non-*H. pylori *helicobacter infection and the presence of *H. pylori *co-infection which precluded assessment of the histological effect of these species under consideration. Also, the significance of coinfection in terms of disease development could not be determined. We could have identified few more cases of non-*H. pylori *helicobacter species by other reported methods used to study non *H. pylori *helicobacter species including fluorescent in situ hybridization (FISH), transmission electron microscopy (TEN) and partial 16S ribosomal sequencing for analyses of the amplified products [12,17].

The implications of this study are that non-*H. pylori *helicobacter species infection occurs in patients with abdominal pain or discomfort similar to *H. pylori *infection. Most of our *H. heilmannii *infections were not associated with contact with animals. This is in contrast to a previous analysis of 125 patients with confirmed *H. heilmannii *infection that showed some 70.3% of the 111 patients had a history of contact with one or more animals [17,18]. All of our patients with non-*H. pylori *infection had endoscopic gastritis though their association with peptic ulcer is well known [19,20]. The prevalence of coinfection of *H. felis *with *H. pylori *in our population is less than what has been reported from South Africa among African population but is certainly higher than that for *H. heilmannii *and *H. pylori *from the northern Europe which showed that only 1.6% had concomitant infection with *H. pylori *[20,21]. The coinfection in our patients demonstrated severe gastric pathology, as intestinal metaplasia was present in 25% of *H. heilmannii *coinfection with *H. pylori *while in 20% of *H. felis *coinfection with *H. pylori*. This was also reported in previous studies [22]. In this study, the difference was not statistically significant due to the number of subjects in each group. The routine transmission of *H. pylori *appears to be human-human whereas non-*H. pylori *helicobacter species are transmitted by cats, dogs, etc [22]. Consequently, the prevalence of *H. heilmannii *is expected to be significantly higher in environment with less hygiene and higher physical exposure to animals. However, in our study there was a negative association with pet contact as the patients reported limited exposure to these animals. There is a need to look into other modes of transmission of these infections.

## Conclusion

As non *H. pylori *Helicobacter species are capable of producing complications similar to *H. pylori *so the identification of these species may be of importance in patients with dyspepsia. However, our study fails to show any increased risk of infection with these organisms on exposure to pet animals and any additional complications associated with co-infection in patients infected with *H. pylori*.

## Methods

### Study population

Between September 2009 and February 2011, a total of 250 patients with abdominal pain or discomfort who attended the gastroenterology outpatient clinic at a tertiary care hospital in Karachi were enrolled. The mean age of these patients was 39 ± 12 years, (range 18-75) with males 162(65%) and females 88(35%). Of these, 136(54%) were in the age group of 18-39 years, 88(35%) in the group of 40-55 years and 26(10%) in the group of 56-75 years. Ethical approval for the study was obtained from the Aga Khan University Ethics Review Committee. Informed consent was taken for participation in the study. A complete socio-demographic questionnaire including determination of socio-economic status, educational level, ownership of the place of residence, number of rooms in the house, number of people living in the household beside siblings, source of water supply e.g. municipal water pipeline or bore water (ground water) and type of latrine in use, was obtained from the patients. A history of exposure of enrolled patients to cats and dogs was determined and a physical examination was carried out. Inclusion criteria were i) ambulatory adult males and non-pregnant females; ii) age 18 years or older; iii) patients with upper GI symptoms including abdominal/epigastric pain or discomfort, postprandial abdominal distension, postprandial nausea and vomiting. Exclusion criteria included i) receiving treatment for *H. pylori*, concurrent or recent antibiotic use such as metronidazole, clarithromycin, amoxicillin, tetracycline, doxycycline and other cephalosporin, ii) histamine-2 receptor blocker or proton pump inhibitor therapy and bismuth compounds in the last four weeks; iii) patients with regular use of NSAID; iii) patients with severe concomitant disease and iv) patients with upper GI surgery. A crowding index with three categories was constructed by dividing the number of individuals per household by the number of the rooms used as bedrooms [23]. A participant's household crowding was defined as 'low' if they scored an index of 0-1.0, moderately-crowded were '2-4' and > 4 were highly 'crowded'.

On EGD, 242(97%) were found to have endoscopic gastritis (GS) alone while 8(3%) had duodenal ulcer (DU). Biopsy specimens from the gastric corpus and antrum were taken for rapid urease test (RUT) or histopathology for the diagnosis of *H. pylori *and DNA extraction for polymerase chain reaction (PCR) to amplify *H. pylori, H. felis *and *H. heilmanii *genes. Ninety patients (36%) out of 250 had a RUT done while 160(64%) out of 250 had histology and provided gastric biopsy specimen for the detection of Helicobacter species.

### Histopathology

Biopsy specimens were stained with hematoxylin and eosin. Sections were examined by an experienced gastrointestinal pathologist blinded to the clinical details of the patients and graded according to the updated Sydney classification [24]. The bacterial density was graded from 0 to 3 (0, absent or occasional; 1 to 3, from few and isolated bacteria to colonies). The infiltration of gastric mucosa by mononuclear cells and polymorphonuclear leucocytes, atrophy, and intestinal metaplasia were graded as follows: 0, none; 1, mild; 2, moderate; 3, marked. Chronic inflammation was defined according to an increase in lymphocytes and plasma cells in the lamina propria graded into mild, moderate or marked increase in density. Chronic active gastritis indicated chronic inflammation with neutrophilic polymorph infiltration of the lamina propria, pits or surface epithelium graded as 0 = nil, mild = < 1/3 of pits and surface infiltrated; moderate = 1/3-2/3; and marked = > 2/3. Gastritis was scored by total sum of grade of gastritis (mild = 1, moderate = 2, marked = 3 infiltration with lymphocytes and plasma cells) and activity of gastritis (mild = 1, moderate = 2, marked = 3 infiltration with neutrophilic granulocytes) either in the antrum or in the corpus. Atrophy was defined as the loss of glandular tissue, with or without replacement by intestinal-type epithelium. Criteria for a true positive result was established with positive RUT or histology and 16S rDNA amplification.

### DNA Extraction

DNA was extracted from biopsy samples by using a QIAamp DNA mini kit from QIAGEN (Hilden, Germany) according to the manufacturer's protocol. Extracted DNA was stored at -70°C until required.

### Polymerase chain reaction

PCR was performed using extracted DNA as the template to identify *H. pylori, H. heilmannii *and *H. felis*. Samples that were positive for Helicobacter genus 16S rDNA were subsequently analyzed with different sets of previously published primers (Table 4) which encode *H. pylori *phosphoglucosamine mutase (*glmM*), *H. heilmannii ureB *and *H. felis *internal fragment of the *ureA *and *ureB *genes, respectively [14,21,25,26]. PCR amplification was carried out in a total volume of 25 μl containing 2 μl of 2 mM dNTPs, 1 μl of 50 ρmol of each forward and reverse primer used before [14,25-27]. (synthesized by MWG Automatic synthesizer, Germany), 2.5 unit of Taq DNA polymerase (Promega, USA), 2.5 μl of 10 × PCR reaction buffer, 3 mM of MgCl_2_, 2 μl of DNA template containing 0.5 ng of extracted DNA and total volume rounded to 25 μl by double distilled water. The reaction was carried out in a Perkin Elmer 9700 thermal cycler (Massachusetts, USA). The amplification cycles for the different Helicobacter spp. gene fragments were: 94°C for 5 min; 94°C for 1 min, 55°C-58°C for 1 min, 72°C for 60--90 sec (35 cycles); 72°C for 5-7 min. Positive and negative reagent control reactions were performed with each batch of amplifications. After PCR, the amplified PCR products were electrophoresed in 2% agarose gels containing 0.5 × Tris/acetate/ethylenediaminetetraacetic acid, stained with ethidium bromide, and visualized under a short wavelength ultraviolet light source. DNA from *H. pylori *strains ATCC 43504, *H. felis *ATCC 49179 and *H. heilmannii *JF804941.1 was used as a positive control and sterile deionized water as the negative control. Diagnosis of each of the *Helicobacter *species infection was established when Helicobacter genus PCR for 16S rDNA was positive along with a species specific PCR for *H. pylori, H. heilmannii *or *H. felis*. PCR product of *H. heilmannii *and *H. felis *were sequenced to further confirm individual infection. The specificity of *H. pylori *phosphoglucosamine mutase (*glmM*) and segment of urease B primers for *H. heilmannii *and *H. felis *has been demonstrated previously [14,21,25-27].

**Table 4:** Oligonucleotide primers used in this study to amplify Helicobacter spp. gene fragments

Gene	Sequence (5' to 3')	Amplicon size (bp)	Reference
**Helicobacter 16S rRNA**			
C97	GCT ATG ACG GGT ATC C	400	18
C 98	GAT TTT ACC CCT ACA CCA		
***H. pylori glmM***			
F	GGATAAGCTTTTAGGGGTGTTAGGGG	294	19
R	GCTTACTTTCTAACACTAACGCGC		
***H. heilmannii ureB***		580	14
F	GGGCGATAAAGTGCGCTTG		
R	CTGGTCAATGAGAGCAGG		
***H. felis ure A and B***		241	20
F	GTG AAG CGA CTA AAG ATA AAC AAT		
R	GCA CCAAAT CTA ATT CAT AAG AGC		

### Sequencing of PCR product and BLAST Query

The DNA fragments amplified by *H. felis *and *H. heilmannii *PCRs were purified by Qiagen quick PCR purification kit (Qiagen, USA) and sequenced using both the forward and reverse primers (Table 4) to verify that they represented truly the *H. felis *and *H. heilmannii ureB *gene. Sequence analysis was performed by Macrogen (Seoul, South Korea). ClustalX was used to edit the sequences. The sequences were edited to a length of 488 bp for *H. heilmannii *and 210 bp for *H. felis*. Homology of the DNA sequences to published sequences was determined by using BLAST window on the National Center for Biotechnology Information (NCBI) site at http://www.ncbi.nlm.nih.gov/BLAST.

### Nucleotide sequence accession numbers

The sequenced PCR products of *H. heilmannii *and *H. felis *obtained in this study have been deposited in GenBank under the following accession numbers: JF804941, JF804942, JF804943, JF804944, JF804945, JF815095, JF815096, JF815097 and JF815098. PCR product sequences were compared to the sequences of Urease B of *H. heilmannii *sequences GenBank: AF508012, L25079.1, AY139171.0, AY139171.1 and *H. felis *strains ref GenBank: FQ 6701792, AY368267.1 and AY368261.1.

### Statistical Method

Using software EPI Info and using 10% prevalence in the study population [21] with 95% confidence level and a bound on error of ± 4% the estimated sample size was 217.

Results are expressed as mean ± standard deviation for continuous variables (e.g., age) and number (percentage) for categorical data (e.g., gender, etc.). Univariate analysis was performed by using the independent sample t-test, Pearson Chi-square test and Fisher Exact test whenever appropriate. A *P*-value of < 0.05 was considered as statistically significant. All p-value were two sided. Statistical interpretation of data was performed by using the computerized software program SPSS version 19.

## Competing interests

The authors declare that they have no competing interests.

## Authors' contributions

JY conceived and designed the study, JY, ZAB, RK, WJ coordinated the study, JY, SN, FJ did the work, JY and ZA analyzed the data, ZAH analyzed the histopathology, JY, MI, SA performed the statistical analysis. JY wrote the manuscript. All authors read and approved the final manuscript.

## Pre-publication history

The pre-publication history for this paper can be accessed here:

http://www.biomedcentral.com/1471-230X/12/3/prepub
